# Quality of nursing documentation before and after the Hospital Accreditation in a university hospital^1^

**DOI:** 10.1590/1518-8345.0686.2813

**Published:** 2016-11-21

**Authors:** Aline Tsuma Gaedke Nomura, Marcos Barragan da Silva, Miriam de Abreu Almeida

**Affiliations:** 2 MSc, RN, Hospital de Clínicas de Porto Alegre, Porto Alegre, RS, Brazil.; 3 Doctoral student, Escola de Enfermagem, Universidade Federal do Rio Grande do Sul, Porto Alegre, RS, Brazil.; 4 PhD, Adjunct Professor, Escola de Enfermagem, Universidade Federal do Rio Grande do Sul, Porto Alegre, RS, Brazil.

**Keywords:** Nursing Records, Nursing Audit, Hospital Accreditation, Education Department, Hospital

## Abstract

**Objective:**

to analyze the quality of nursing documentation by comparing the periods before and after the preparation for the hospital accreditation, using the Quality of Nursing Diagnoses, Interventions and Outcomes - Brazilian version (Q-DIO- Brazilian version).

**Method:**

observational study of interventions conducted in a university hospital. Nursing documentation of 112 medical records for the period before and 112 for the period after the hospital accreditation were compared using the Q-DIO instrument - Brazilian version. Data were statistically analyzed.

**Results:**

there was a significant improvement in the quality of nursing documentation. When the total score of the instrument was evaluated, a significant improvement was observed in 24 out of the 29 items (82.8%).

**Conclusion:**

there was commitment to the shift of culture by means of the interventions carried out, which resulted in the conquest of the quality seal ensured by the Joint Commission International.

## Introduction

The major purpose of the hospital accreditation is to create a culture of safety and quality in the institutions interested in continually improving patient care processes^1^. In this context, it is worth pointing out that electronic health records are relevant information sources to monitor the achieved quality and safety levels^2^.

This operationalization is in line with the principles of the institution where the study was carried out. In this scenario, the nurses have used the information technology as a tool for promoting quality in health care management, especially for clinical decision making in the Nursing Process (NP)^1^.

In order to adhere to the international accreditation, various strategies aiming to obtain the quality seal were employed in the hospital, resulting in the recognition in 2013. To achieve this goal, it was sought an organization based on the criteria described in the Accreditation Standards Manual of the *Joint Commission International* (JCI) for hospitals^3^.

From the creation of this manual, the records become an important focus of evaluation in institutions, both at organizational and assistance levels. Consequently, the quality of computerized nursing documentation assumes a position of great importance for achieving the hospital accreditation. This manual is divided into two sections: *Section I - Patient-Centered Care Standards,* composed of eight evaluative items and *Section II - Health Facilities Management Standards,* composed of six evaluative items^1^. Ten out of the fourteen items evaluated by the JCI are directly related to the nursing documentation, that is, all items of section I and two of section II include in their assessment, the documentation performed by the nursing team in the patient medical record.

In this evaluation context, the nursing team should carry out records that reflect the patient care in a clear and reliable fashion, considering a new professional approach in response to technological advances, globalization and the change required in the workplace^4^. In this way, only having a computerized system does not ensure the completeness and quality of records^5^, requiring assessments and improvements in a systematic way.

Within this perspective is the instrument *Quality of Nursing Diagnoses, Interventions and Outcomes - Brazilian version* (Q-DIO- Brazilian version) validated for the Portuguese language to assess the quality of nursing documentation^6^ ^-^ ^7^. This has been used as an indicator to assess and compare the quality of these records and used as a tool in auditing systems and in the assessment of the impact of the implemented educational programs^7^.

In order to assess the changes in quality of computerized nursing documentation, this study evaluated this quality at two different moments, before and after the preparation for the hospital accreditation by means of the Q-DIO - Brazilian version.

As justification of the study, it is believed that this will encourage hospitals to establish goals and improvements in care, aiming at the quality of nursing documentation, through interventions to increase the commitment of the institution in favour of quality and safety of health care provided.

In this sense, it was intended to answer the following research question: Did the intervention developed during the hospital accreditation process help to improve the quality of nursing documentation?

## Objetive

To analyze the quality of nursing documentation by comparing the periods before and after the preparation for the hospital accreditation and using the Q-DIO - Brazilian version.

## Method

This is an observational study of interventions, which can be used when there is no consensus about the efficiency of a single activity implemented; thereby, different activities can be carried out regardless of the quality of the evidences. The Intervention consisted of actions performed by nurses in the period of preparation for the Hospital Accreditation, aiming at improving the quality of nursing documentation on the patient history. These Interventions consisted of clinical case studies; printed bulletins and electronic newsletters; regular support group meetings; theoretical and practical training; development of brochures and manuals; restructuring of the computerized documentation; Anamnesis and nursing physical examination, including information on the education of the patient and relatives and obligatory assessment items; provision of a specific space for training, disclosure of information and clarification of doubts; development of an institutional list of abbreviations or acronyms; creation of focused groups and active and educational visits in inpatient units; implementation of educational evaluations and establishment of strategies to remedy or alleviate the problems encountered; training lectures; development and revision of Standard Operating Procedures; development of Distance Learning courses.

The study site was a general and public university hospital, which serves 60 specialties and has approximately 850 beds shared among 10 Nursing Services. The NP used as a working method has the ND stage based on the terminology of the NANDA International (NANDA-I) and the prescribed care based on the Nursing Interventions Classification (NIC)^8^.

The study sample consisted of nursing documentation of patients hospitalized in the clinical and surgical inpatient units in the predetermined period.

The sample was calculated using PEPI software (*Programs for Epidemiologists*) version 4.0 and based on the study "Validation of the Q*uality of Diagnoses, Interventions and Outcomes* (Q-DIO) for use in Brazil and the United States of America"^7^ and on the number of hospitalizations in October 2013 in the units of interest, totaling 1,018 admissions. Considering a confidence level of 95%, a standard deviation of 4.5 on the Q-DIO score - Brazilian version and a margin of error of 3%, a minimum of 224 records was required, 112 for each year, 52 of these were from clinical units and 60 were from surgical units. Inclusion criteria were the records of patients hospitalized for at least four days in the same hospital unit. Exclusion criteria were not set out. To obtain the sample, 2,182 records were selected through a *query* (mechanism for retrieving information from a database in an electronic system) and randomized. All clinical and surgical inpatient units were considered proportionally to the number of hospitalizations for the period.

Data collection was carried out by a master's student and a doctoral student of the university postgraduate program linked to the hospital where the study was conducted. The researchers were trained for completion of the Q-DIO - Brazilian version by one of the authors of the instrument.

To estimate the correlation between the two evaluators in completing the Q-DIO - Brazilian version, a pilot study was conducted with 10% of the sample (24 clinical records), with excellent compliance for the four domains.

The data collection instrument used was the Q-DIO - Brazilian version. Its structure consists of four areas: *Nursing diagnosis as process,* assessed on the basis of 11 items, *Nursing diagnosis as a product*, assessed on the basis of 8 items, *Nursing interventions,*, assessed on the basis of 3 items, e and *Nursing outcomes,* assessed on the basis of 7 items, using a 3-point Likert scale. The Q-DIO - Brazilian version was computerized by using a tool developed through a WEB application, and whose interface was created in HTML format and ASP programming language. Data were collected between December 2013 and January 2014, from computerized and printed documentation. Data collection was retrospective and refers to two moments, during the month before and the last month of the preparation for the Accreditation, that is, October 2009 and October 2013. The 224 records were randomly selected.

Data analysis was performed by comparing the total score of *Q-DIO* instrument - Brazilian version, considering the four domains and their respective questions. The Mann-Whitney test was used to compare the asymmetric continuous variables, which were represented by median and interquartile range. The comparison between categorical variables was performed using the chi-square test and the results were represented by absolute values and percentages, with adjusted standardization of residues and, when indicated, Fisher's exact test was used. The level of significance used was 5% (p<0.05). Statistical analysis was performed with the *Statistical Package for the Social Sciences* software (SPSS), version 18.0 for Windows.

The research project followed all ethical procedures necessary to the approval by the Research Ethics Committee of the institution, under protocol number 130389.

## Results

The total score of the instrument Q-DIO - Brazilian version showed a significant difference between the years, considering a minimum score of "zero" and a maximum of 58 points. Based on the assessment of 112 records for each year, the median and interquartile range increased from 31 (28-37) in 2009 to 43 (37-47) in 2013.

There was a significant difference among the four domains, with an emphasis on the maximum score of 22 points in the first domain, 16 points in the second, six in the third and 14 in the last domain, according to data shown in Table 1.

**Table 1:** Comparison of the domains of Q-DIO instrument - Brazilian version, between 2009 and 2013 in the overall sample (n=224). Porto Alegre, RS, Brazil, 2014

Questions	2009 Md (P25 - P75)	2013 Md (P25 - P75)	p
Domain of Nursing diagnoses as process	5 (2 - 9)	13 (9 - 16)	<0.001
Domain of Nursing diagnoses as product	15 (13 - 16)	15 (14 - 16)	0.002
Domain of Nursing Interventions	6 (5 - 6)	6 (6 - 6)	<0.001
Domain of Nursing Outcomes	7 (4 - 9)	9 (7 - 11)	<0.001

Data represented by median and interquartile range

When each question of the first domain was evaluated, considering the three categories of records classification ("zero" for *undocumented*, 1 for *partially documented* and 2 for *complete documentation*), it was observed that there was an improvement of documents, reducing the proportion of records with the value "zero" and increasing the proportions of the other categories, as shown in Table 2.

**Table 2:** Comparison of the scores of the Domain Nursing Diagnoses as process of Q-DIO - Brazilian version, between 2009 and 2013 in the overall sample (n=224). Porto Alegre, RS, Brazil, 2014

Questions	Years	0	1	2	P
1. Actual situation leading to the hospitalization	2009	13 (11.6)	37 (33.0)	62 (55.4)	<0.001
	2013	1 (0.9)	18 (16.1)	93 (83.0)	
2. Anxiety and worries related to hospitalization, expectations and desires about hospitalization	2009	54 (48.2)	31 (27.7)	27 (24.1)	<0.001
	2013	18 (16.0)	47 (42.0)	47 (42.0)	
3. Social situation and living environment/circunstances	2009	67 (59.8)	31(27.7)*	14 (12.5)	<0.001
	2013	37 (33.0)	44(39.3)	31 (27.7)	
4. Coping situation and living environment/circunstances	2009	64 (57.1)	31 (27.7)	17 (15.2)	<0.001
	2013	19 (17.0)	49 (43.8)	44 (39.2)	
5. Beliefs and attitudes about life (related to the hospitalization)	2009	93 (83)	19 (17)	0 (0)	<0.001
	2013	27 (24.1)	50 (44.6)	35 (31.3)	
6. Information of the patient and relatives/significant others about the situation	2009	106 (94.6)	6 (5.4)	0 (0)	<0.001
	2013	23 (20.5)	84 (75)	5.0 (4.5)	
7. Issues about personal intimacy related to gender	2009	89 (79.5)	14 (12.5)	9 (8.0)	0.665
	2013	86 (76.8)	13 (11.6)	13 (11.6)	
8. Hobbies, activities for leisure	2009	81 (72.3)	24 (21.4)*	7 (6.3)	<0.001
	2013	46 (41.1)	36 (32.1)	30 (26.8)	
9. Significant others (contact persons)	2009	85 (75.9)	14 (12.5)*	13 (11.6)	<0.001
	2013	20 (17.9)	24 (21.4)	68 (60.7)	
10. Activities of daily living	2009	70 (62.5)	38 (33.9)	4 (3.6)	<0.001
	2013	29 (25.9)	61 (54.5)	22 (19.6)	
11. Relevant nursing priorities according to the assessment	2009	13 (11.6)	55 (49.1)	44 (39.3)	<0.001
	2013	0 (0)	22 (19.6)	90 (80.4)	

Data represented by absolute values and percentages.

* Non-significant residual analysis.

By evaluating the differences in each assignment category of questions between the years, it was observed that there were no statistical differences in the *Questions 3, 8* and *9* of the category 1, although there were more than 10% increase between the years.

As for the domain *Nursing diagnoses as product*, considering the differences in the records assessment categories between the years, no statistical difference was observed in category 1 on nearly all questions, except in *Question 18*. It is observed a small amount of records in the category "zero", in contrast to a large amount in the category 2 in the assessed years, as shown in Table 3.

**Table 3:** Comparison of the scores of the domain Nursing diagnoses as product of Q-DIO - Brazilian version, between 2009 and 2013 in the overall sample (n=224). Porto Alegre, RS, Brazil, 2014

Questions	Years	0	1	2	p
12. Nursing problem/ Nursing diagnosis is documented	2009	7 (6.3)	1 (0.9)*	104 (92.8)	0.036
	2013	1 (0.9)	0 (0)	111 (99.1)	
13. Nursing diagnosis label is formulated and numbered according to NANDA.	2009	7 (6.3)	1 (0.9)*	104 (92.8)	0.036
	2013	1 (0.9)	0 (0)	111 (99.1)	
14. The etiology is documented	2009	7 (6.3)	1 (0.9)*	104 (92.8)	0.036
	2013	1 (0.9)	0 (0)	111 (99.1)	
15. The etiology is correct, related and corresponding to the nursing diagnosis	2009	7 (6.3)	8 (7.1)*	97 (86.6)	0.046
	2013	1 (0.9)	5 (4.5)	106 (94.6)	
16. Signs and symptoms are documented	2009	8 (7.1)	25 (22.3)*	79 (70.6)	0.023
	2013	2 (1.8)	15 (13.4)	95 (84.8)	
17. Signs and symptoms are correctly related to the nursing diagnosis	2009	9 (8.0)	24 (21.4)*	79 (70.6)	0.018
	2013	2 (1.8)	15 (13.4)	95 (84.8)	
18. The nursing goal relates/corresponds to the nursing diagnosis	2009	12 (10.7)	66 (58.9)	34 (30.4)	0.002
	2013	4 (3.6)	50 (44.6)	58 (51.8)	
19. The nursing goal is achievable through nursing interventions	2009	10 (8.9)	21 (18.8)*	81 (72.3)*	0.087
	2013	3 (2.7)	17 (15.2)	92 (82.1)	

Data represented by absolute values and percentage.

* Non-significant residual analysis.

As for the domain Nursing Interventions, in the assessment of the differences in each classification category of records between the years, it was observed that the same *Question 20* showed no difference in any of the categories, whereas *Question 21* showed a significant difference only in the category "zero" and *Question 22* showed no significant difference in the category "zero". It is emphasized, however, a large amount of records in the category *Full Documentation* both in 2009 and 2013, according to Table 4.

**Table 4:** Comparison of the scores of the domain Nursing Interventions of Q-DIO - Brazilian version, between 2009 and 2013 in the overall sample (n=224). Porto Alegre, RS, Brazil, 2014

Questions	Years	0	1	2	p
20. Concrete, clearly named according to Nursing Interventions Classification (NIC) - and planned (what will be done, how, how often, who does it)	2009	1 (0.9)*	1 (0.9)*	110 (98.2)*	0.499
	2013	0 (0)	1 (0.9)	111 (99.1)	
21. The nursing interventions affect the etiology of the nursing diagnosis	2009	8 (7.1)	11 (9.9)*	93 (83.0)*	0.033
	2013	1 (0.9)	9 (8.0)	102 (91.1)	
22. Nursing interventions carried out are documented (what was done, how, how often, who did it)	2009	1 (0.9)*	22 (19.6)	89 (79.5)	<0.001
	2013	0 (0)	5 (4.5)	107 (95.5)	

Data represented by absolute values and percentage.

* Non-significant residual analysis.

In assessing the classification categories, it was observed that there was no statistical difference in the *Question 23* for the categories "zero" and 1 and for the three categories of *Question 26*. Furthermore, there was no difference between the years in category 1 regarding the *Questions 28* and *29*. The results of the fourth domain of Q-DIO - Brazilian version are shown in Table 5.

**Table 5:** Comparison of the scores of the domain Nursing Outcomes of Q-DIO - Brazilian version, between 2009 and 2013 in the overall sample (n=224). Porto Alegre, RS, Brazil, 2014

Questions	Years	0	1	2	P
23. Acute changing diagnosis are assessed daily or form shift to shift/ enduring diagnoses are assessed every four days	2009	8 (7.1)*	29 (25.9)*	75 (67.0)	0.057
	2013	3 (2.7)	19 (17.0)	90 (80.3)	
24. The nursing diagnosis is reformulated	2009	23 (20.5)	38 (34)	51 (45.5)	<0.001
	2013	4 (3.6)	15 (13.4)	93 (83.0)	
25. The nursing outcome is documented	2009	48 (42.8)	63 (56.3)	1 (0.9)	<0.001
	2013	17 (15.2)	87 (77.7)	8 (7.1)	
26. The nursing outcome is observably/measurably documented according to NOC	2009	112 (100)*	0 (0)*	0 (0)*	0.498 ^(F)†^
	2013	110 (98.2)	2 (1.8)	0 (0)	
27. The nursing outcome shows: - improvement in patient's symptoms; - improvement of patient's knowledge; - improvement of patient's coping strategies; - improved sef-care abilities; - improved functional status	2009	85 (75.9)	14 (12.5)	13 (11.6)	<0.001
	2013	37 (33.0)	44 (39.3)	31 (27.7)	
28. There is a relationship between outcomes and nursing interventions	2009	20 (17.9)	39 (34.8)*	53 (47.3)	0.005
	2013	6 (5.4)	34 (30.4)	72 (64.2)	
29. The nursing outcomes and nursing diagnoses are internally related	2009	17 (15.2)	38 (33.9)*	57 (50.9)	0.005
	2013	4 (3.6)	34 (30.4)	74 (66.0)	

Data represented by absolute values and percentage.

* Non-significant residual analysis.

† Fisher's exact test

In this way, there was a significant improvement in almost all items (24/29=82.8%) of Q-DIO - Brazilian version, considering the period before and after the preparation for Hospital Accreditation.

## Discussion

Hospital Accreditation encouraged the planning and implementation of interventions capable of resulting in the improvement of nursing documentation. In a cross-sectional study, it was found that nurses from hospitals accredited to the accreditation were more receptive to changes and improvements when compared to the non-accredited hospitals. In this perspective, this assessment process motivates professionals to organize themselves to better understand the compreensive patient care^9^.

Considering the total score and the four domains of Q-DIO - Brazilian version, it is clear that there was a significant overall improvement of records between the years evaluated. Among the domains of Q-DIO - Brazilian version, which showed the most representative improvement was the *Nursing Diagnosis as Process*. Its questions involve individual and overall assessment to identify relevant nursing information in the records during the first 24 hours of the patient hospitalization^7^. This information is usually found in the document Nursing Anamnesis, in which it is possible to know the customer, establish links, identify the biopsychosocial and spiritual changes, and thus define the nursing diagnoses, expected outcomes and interventions that enable the patient improvement^10^.

As regards the questions of the first domain of Q-DIO - Brazilian version, most of them showed a significant improvement in quality between the years evaluated. It is inferred that this result was partly due to the restructuring of the Anamnesis and Nursing Physical Examination in the electronic information system. It should be noted that the assessment of questions 5 and 6 was not observed in 2009, being regarded in 2013. It is believed that systematically operationalize the existing tools, in association with the continuing education of nurses, has a positive influence on the quality of nursing documentation. It is highlighted the need for patient care under biological, psychosocial and spiritual aspects, rescuing a holistic view on this patient^10^.

The inadequacy of *Question 7* may have been a result of the unpreparedness of the nurses to assess gender and sexuality issues. It is highlighted the lack of studies on sexuality in the training of nurses, despite its importance in overcoming the heteronormativity and considering the new marital patterns and family arrangements^11^.

As regards the Nursing Evolution, the domain *Nursing diagnosis as product* assesses the nursing documentation regarding the individual situation of the patient according to the format Problem, Etiology, Signs and Symptoms (PES), nursing diagnoses and goals^6^. The evolution model adopted in the institution contains the items: subjective, objective, interpretation and practices, including those related to patient education. Since the system used is computerized and uses the terminology of the NANDA-I^8^, *Questions 12, 13* and *14* did not reach the maximum score only when there was no daily Nursing Evolution, being dispensable in this assessment. The significant improvement in *Question 15* is also due to the clinical judgment of nurses in choosing accurate etiologies.

With regard to the *Questions 16* and *17*, a significant improvement was observed, suggesting a critical development of nurses in choosing nursing diagnoses in the register of their respective defining characteristics. The registration of such characteristics was contained in the subjunctive and/or objective of the nursing evolution, supporting the listed diagnoses.

*Question 18* assesses the quality of nursing conduct and showed significant improvement in the period evaluated. However, this registration practice needs to be strengthened, since it corresponded to a representative portion of the category "*partly documented*", with 58.9% in 2009 and 44.6% in 2013. The registration of nursing outcomes is not yet a reality in the institution, although its application in clinical practice have been investigated in the evolution of patients^8^.

*Question 19* of this domain did not show significant improvement between the assessed groups, although this criterion presented 72.3% compliance in 2009, increasing to 82.1% in 2013. Since the nursing objectives at the study institution were not clearly named, it was sought to assess whether the nursing prescription contained the specific care capable of solving the identified nursing problems.

The domain *Nursing Intervention* showed a significant improvement between the years. Among the questions, only *Question 20* showed no significant difference*.* Despite this result, it appears that 110 records performed in 2009 showed compliance, and 111 in 2013, rates resulting from automation and systematic operationalization of records.

The significant improvement in *Question 21* suggests the presence of critical thinking and clinical judgment of nurses in assessing the patient's specific needs in order to prescribe a plan of care, preserving their individuality. A study shows that there is still little effectiveness in the application of scientific knowledge in the development of individualized care for each patient^12^. However, it can be noted that the nurses of the institution have improved their ability to individualize nursing interventions.

The *Question 22* demonstrated significant improvement, indicating that the care check has become more valued in the institution. On the other hand, in a study that assessed the check of nursing prescriptions, found that out of the 70.7% of records present, only 1% of the prescriptions was completely checked^13^.

Among the items of the domain *Nursing Outcomes*, the *Question 23* showed no significant improvement in quality between the years evaluated. However, when the classification category was assessed between the years, it was noticed a significant improvement in category 2, "Full documentation", increasing from 67% to 80.4%.

As for the significant improvement in quality of *Question 24* between the years, it was shown that nursing diagnoses have been better registered and/or reformulated in the final year of the educational process for the Accreditation. However, studies have shown inconsistency regarding the completeness of the phase of nursing diagnosis^13^ ^-^ ^14^. Although the institution where the study was conducted has not included the *Nursing Outcomes Classification* (NOC) in the NP^15^ up to this moment, *Question 25* also showed a significant improvement. This is due to the more frequent use of the terms "improved", "worsened", "maintained" and "resolved" followed by the nursing diagnosis and its etiology. However, most medical records assessed on this item were categorized as "*partly documented*", with 56.3% in 2009 and 77.7 in 2013. *Question 26* showed no significant changes, since the institution is still on a process of research for the effective implementation of NOC in its working method. Studies have shown the applicability of NOC, aiming at the improvement and significant progress not only in the quality of documentation but also in the nursing practices^8^ ^-^ ^16^. Based on these results, it is clear that the operationalization of NOC in the institution tends to benefit the quality of nursing documentation, facilitated by the articulation of standardized languages and computerised systems^15^. Educational strategies for training of nurses will be essential for the implementation of this stage.

The Question 27 showed significant improvement, as well as questions 28 and 29. The presence of these records suggests an improvement on the patient needs outcomes, indicating that the activities performed were effective on the patient care outcomes. However, considering the results of these items, full documentation is still incipient, as even with a significant improvement in the classification category 2, between the years; the rates obtained in 2013 still need to be increased, with 27.7%, 64.3% and 66.1%, respectively, as the maximum scores.

It is observed, therefore, a strong influence of NP in the Hospital Accreditation process, as the record is an important evidence of the quality of care provided to patients and relatives. Therefore, it is necessary to reorganize the work processes through continuous training of the nursing staff in order to promote the appreciation and adherence to the standards recommended by the institution^1^, as well as the monitoring of this process through well-defined instruments.

In this way, it is clear that the Intervention is an influential strategy in the valuation of nurses about the presence and completeness of these records. However, a study using interrupted time series analysis showed that the quality performance decreased after the evaluative stage of accreditation^17^. Thus, management mechanisms with a view to assuring the quality and safety of care should be implemented on a permanent basis and assessed in order to strengthen the safety culture in the institution and evidenced in the records to maintain the quality seal of the JCI.

## Conclusion

In analyzing the quality of nursing documentation through the Q-DIO - Brazilian version, by comparing the period before and after the preparation for Hospital Accreditation, it was found that there was an improvement in the quality of these records. It is concluded that the Intervention led to a significant improvement in almost all criteria assessed.

Based on these results, it can be stated that there was commitment to the culture of change by means of the organizational innovation, implementation of protocols, maintenance of internal audits and, especially, educational activities brought about by the careful evaluation of the JCI. This favored the recognition of the institution as an academic center of excellence for health quality and patient safety by the *Joint Commission International* in 2013. However, since the study was conducted in adult inpatient units, the results are limited to that scenario. Thus, its implementation elsewhere would favor the overall assessment of this quality.

The development of systematic interventions proved to be a strong ally in in the pursuit of international recognition of the quality of care through the Hospital Accreditation, which may help other institutions in the improvement and optimization of nursing documentation.
